# The development of allergic inflammation in a murine house dust mite asthma model is suppressed by synbiotic mixtures of non-digestible oligosaccharides and *Bifidobacterium breve* M-16V

**DOI:** 10.1007/s00394-015-0928-8

**Published:** 2015-05-24

**Authors:** K. A. T. Verheijden, L. E. M. Willemsen, S. Braber, T. Leusink-Muis, P. V. Jeurink, J. Garssen, A. D. Kraneveld, G. Folkerts

**Affiliations:** Division of Pharmacology, Department of Pharmaceutical Sciences, Faculty of Science, Utrecht University, Universiteitsweg 99, 3584 CG, Utrecht, The Netherlands; Division of Veterinary Pharmacy, Pharmacology and Toxicology, Faculty of Veterinary Sciences, Utrecht University, Utrecht, The Netherlands; Nutricia Research, Immunology, Utrecht, The Netherlands

**Keywords:** House dust mite, Asthma, Allergy, Oligosaccharides, *Bifidobacterium breve*

## Abstract

**Purpose:**

The incidence and severity of allergic asthma is rising, and novel strategies to prevent or treat this disease are needed. This study investigated the effects of different mixtures of non-digestible oligosaccharides combined with *Bifidobacterium breve* M-16V (*BB*) on the development of allergic airway inflammation in an animal model for house dust mite (HDM)-induced allergic asthma.

**Methods:**

BALB/c mice were sensitized intranasally (i.n.) with HDM and subsequently challenged (i.n.) with PBS or HDM while being fed diets containing different oligosaccharide mixtures in combination with *BB* or an isocaloric identical control diet. Bronchoalveolar lavage fluid (BALF) inflammatory cell influx, chemokine and cytokine concentrations in lung homogenates and supernatants of ex vivo HDM-restimulated lung cells were analyzed.

**Results:**

The HDM-induced influx of eosinophils and lymphocytes was reduced by the diet containing the short-chain and long-chain fructo-oligosaccharides and *BB* (FF*BB*). In addition to the HDM-induced cell influx, concentrations of IL-33, CCL17, CCL22, IL-6, IL-13 and IL-5 were increased in supernatants of lung homogenates or BALF and IL-4, IFN-γ and IL-10 were increased in restimulated lung cell suspensions of HDM-allergic mice. The diet containing FF*BB* reduced IL-6, IFN-γ, IL-4 and IL-10 concentrations, whereas the combination of galacto-oligosaccharides and long-chain fructo-oligosaccharides with *BB* was less potent in this model.

**Conclusion:**

These findings show that synbiotic dietary supplementation can affect respiratory allergic inflammation induced by HDM. The combination of FF*BB* was most effective in the prevention of HDM-induced airway inflammation in mice.

## Introduction

Allergic asthma is a chronic disease that affects around 235 million people worldwide. Asthma is not only a health problem for developed countries but also for developing countries, and the prevalence is still increasing [1]. The disease is characterized by impaired lung function as well as airway inflammation containing high numbers of eosinophils [2]. House dust mite (HDM) is one of the well-known allergens that can trigger allergic diseases such as asthma [3]. The inflammatory response, mainly the recruitment of eosinophils to the airway tissue and the production of cytokines and chemokines, is initiated by HDM-allergen-specific Th2 cells [4]. During sensitization, allergens can directly trigger or stimulate airway epithelial cells, which subsequently release different cytokines (e.g., IL-33) and chemokines (e.g., CCL20) [5]. In response to IL-33, group 2 innate lymphoid cells (ILC2) will proliferate and produce IL-4, -5 and -13 [6]. In addition, IL-33 also induces the maturation of dendritic cells (DC) having Th2-inducing polarizing capacities [7]. These specific DCs release Th2-inducing chemokines such as CCL17 and CCL22. The activated DCs take up the HDM allergen and induce the development of naïve T cells into antigen-specific Th2 cells in the local lymph nodes. Subsequently, these Th2 cells migrate back to the lung mucosal tissue. After encountering DC presenting the antigen in the airways, these effector Th2 cells will release IL-4, IL-5 and IL-13 [5], which can trigger allergic symptoms and eosinophilic inflammation [8]. Novel preventive and/or therapeutic approaches are needed to prevent and/or treat asthmatic disorders, while current treatment is still not sufficient and has considerable side effects. Results from animal and human studies suggest that changes in the intestinal microbiota can contribute to the development of asthma. Indeed different animal studies have shown that the gut microbiota affects systemic immune function [9–12]. Since the composition of the microbiota is important for a balanced immune response, adapting the microbiota using oligosaccharides either or not combined with beneficial bacteria may help to protect against the development of allergic disease. For example, treatment with *B.breve* M-16V was found to suppress airway inflammation and treatment with *Lactobacillusrhamnosus* also reduced lung resistance in a murine ovalbumin-induced asthma model [13]. In addition to the direct use of probiotics, also specific non-digestible oligosaccharides such as galacto-oligosaccharides (GOS) and short-chain and long-chain fructo-oligosaccharides (scFOS and lcFOS, respectively) can be administered to support growth and/or activity of bifidobacteria and lactobacilli [9, 14, 15]. In previous studies, we have shown that GOS alone are capable of suppressing HDM-induced airway hyperresponsiveness, airway eosinophilia and Th2-related cytokine and chemokine concentrations in the lung [16]. Furthermore, the combination of GF*BB* or FF*BB* with acidic oligosaccharides reduced allergic responses in food allergic mice and suppressed airway inflammation in a mouse model for ovalbumin-induced asthma, respectively [17, 18]. In mice affected with food allergy, GF*BB* was more effective than GF or *BB* alone [17]. When GF*BB* was used in patients suffering from HDM-induced allergy and asthma, the peak expiratory flow was increased and the production of systemic Th2 cytokines (IL-4, IL-5 and IL-13) was reduced. However, in this study, no effect on bronchial allergic inflammation was demonstrated [19]. GOS are prepared from lactose derived from cow’s milk. Fructo-oligosaccharides, obtained from chicory root, provide a vegetable source of prebiotic oligosaccharides and may be used as an alternative for GOS in the synbiotic mixture. The aim of this study was to investigate the effect of two different synbiotic mixtures, GF*BB* and FF*BB*, on pulmonary inflammation, cytokine and chemokine concentrations in lungs and BALF of HDM-induced asthmatic mice.

## Materials and methods

### Mice

Six- to eight-week-old, specific pathogen-free, male BALB/c mice (Charles River, The Netherlands) were housed under bio-contained sterile conditions using HEPA^®^-filtered isocages^®^ (Tecniplast, Italy). Food and water were provided ad libitum. All animal experiments were conducted in compliance with the Guidelines of the Ethical Committee on the Use of Laboratory Animals of the Utrecht University (DEC 2013.II.01.003).

### Murine HDM-induced asthma model

**Fig. 1:**
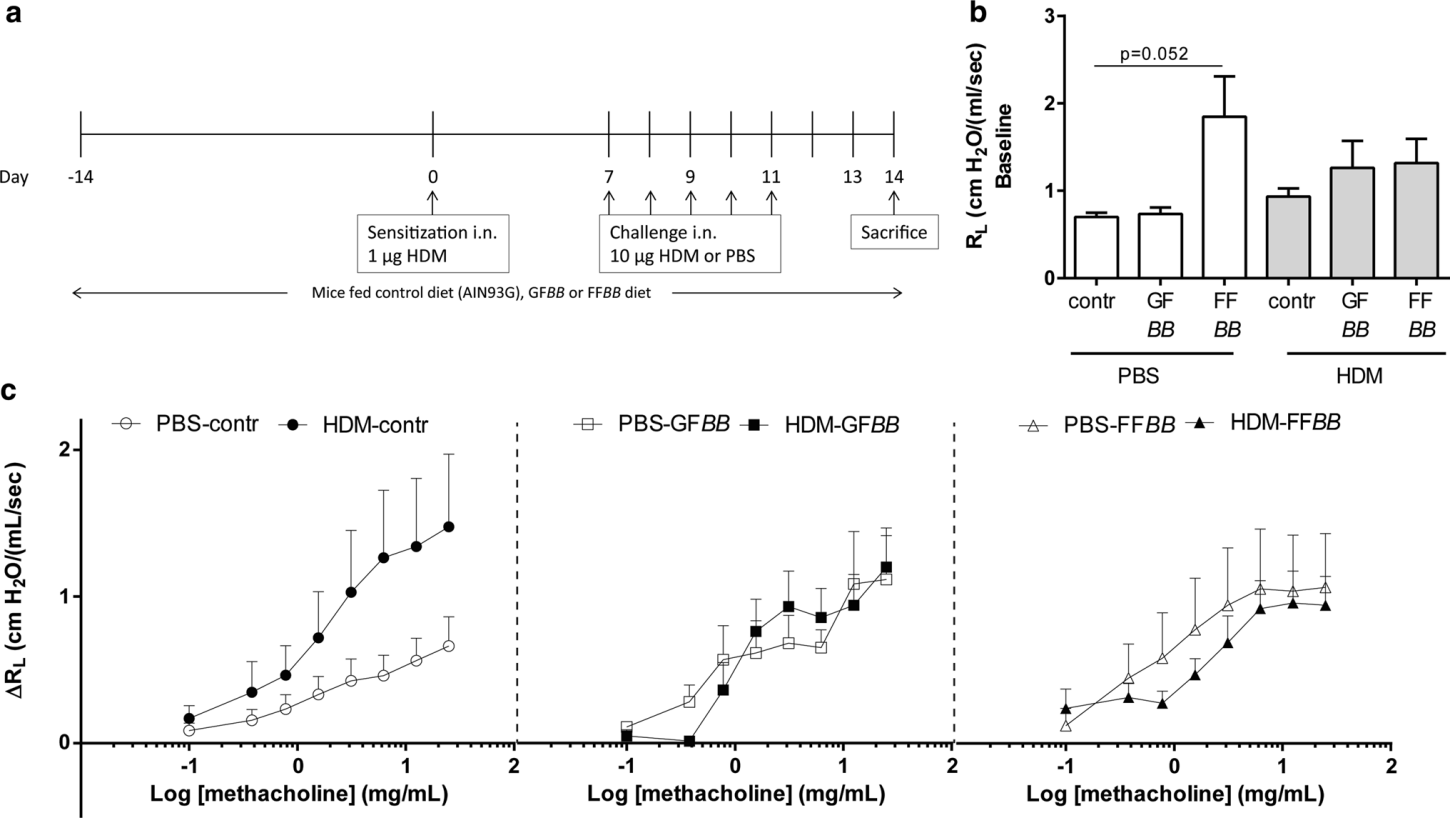
Treatment protocol of the allergic house dust mite (HDM) asthma model and lung resistance measurement. Male BALB/c mice were sensitized intranasally (i.n.) with HDM on day 0 and were challenged i.n. for five consecutive days with HDM or PBS. Mice were fed the control diet (AIN93G, contr), a diet containing a 1 % w/w 9:1 mixture of GOS and long-chain fructo-oligosaccharide (GF) or 1 % w/w 1:1 mixture of short-chain fructo-oligosaccharide and long-chain fructo-oligosaccharide (FF) both in combination with 2×10E9 colony-forming units/g *Bifidobacterium breve* M-16V (*BB*) (2 % w/w). The GF*BB* and FF*BB* interventions started 2 weeks prior to sensitization and continued during the entire experiment. All mice were killed on day 14 (**a**). Basal airway resistance (R_L_) (**b**) and ΔR_L_ after baseline correction in response to increasing doses of methacholine were measured on day 14 (**c**). HDM–PBS: HDM-sensitized and PBS-challenged mice (*white bars*), HDM–HDM: HDM-sensitized and HDM-challenged mice (*gray bars*), Contr: control diet, GF*BB*: mixture of GOS and long-chain fructo-oligosaccharide with *Bifidobacterium breve* M-16V diet, FF*BB*: mixture of short-chain fructo-oligosaccharide and long-chain fructo-oligosaccharide with *Bifidobacterium breve* M-16V diet

While under isoflurane anesthesia, BALB/c mice were intranasally (i.n.) sensitized with 1 µg HDM/40 µL PBS (Greer Laboratories, USA) on day 0 and challenged once a day for 5 consecutive days (day 7–11) with PBS (phosphate buffered saline, control, HDM–PBS) or 10 µg HDM/40 µL PBS (HDM–HDM) [20]. Mice were ad libitum fed the control diet (Research Diet Services, The Netherlands; AIN93G, contr), a diet containing a 1 % w/w 9:1 mixture of GOS (Vivinal^®^ GOS syrup with approximately 59 % GOS, 21 % lactose, 19 % glucose and 1 % galactose on dry matter (dry matter of 75 %); FrieslandCampina Domo, The Netherlands) and long-chain fructo-oligosaccharides (lcFOS, Orafti^®^ HP with approximately ~100 % inulin, DP ≥23; Beneo, Belgium; GF) or a 1 % w/w 1:1 mixture of short-chain fructo-oligosaccharides (Orafti^®^ P95, with approximately 95 % oligofructose content, DP 2–8; Beneo, Belgium) and long-chain fructo-oligosaccharides (FF). Carbohydrates in Vivinal^®^ GOS were compensated isocalorically in the control diet by means of exchange against cellulose (for GOS), lactose (for lactose) and dextrose (for glucose). In case of the fructo-oligosaccharides, carbohydrates were compensated isocalorically in the control diet by means of exchange against cellulose (for FOS). Both GF and FF diets contained 2×10E9 colony-forming units/g (2 % w/w) *Bifidobacterium breve* M-16V (*BB*) (Morinaga milk Industry, Japan). The GF*BB* and FF*BB* diets were given 2 weeks prior to sensitization and were continuously provided throughout the entire experimental period. Mice were killed on day 14 (see treatment schedule in Fig. 1a).

### Airway responsiveness measurement

Mice were anaesthetized with a mixture of ketamine (Vetoquinol S.A., France; 125 mg/kg) and medetomidine (Pfizer, The Netherlands; 0.4 mg/kg), intraperitoneally (i.p.). EMKA invasive measurement of dynamic resistance (EMKA Technologies, France) in response to increasing doses of methacholine (acetyl-β-methyl-choline chloride, Sigma-Aldrich, The Netherlands; 0–25 mg/mL, 10 % puff for 10 s.) was used to determine the lung function on day 14 [13]. Basal airway resistance values of each individual mouse as measured prior to methacholine exposure were deducted from the resistance as measured upon methacholine exposure (ΔR; Fig. 1b–c).

### Bronchoalveolar lavage

Mice were killed on day 14 using an intraperitoneal overdose of pentobarbital (600 mg/kg, Nembutal™, Ceva Santé Animale, The Netherlands). Lungs were lavaged with 1 mL of pyrogen-free saline (0.9 % NaCl, 37 °C) supplemented with protease inhibitor cocktail tablet (Complete Mini, Roche Diagnostics, Germany). The supernatant of the first mL was used for cytokine and chemokine analyses, followed by three lavages with 1 mL saline solution (0.9 % NaCl, 37 °C). The BALF cells were centrifuged (400 g, 5 min), and pellets of the four lavages were pooled; total numbers of BALF cells were counted using a Bürker-Türk chamber (magnification 100×). Cytospin were stained with Diff-Quick (Merz & Dade A.G., Switzerland) for differential BALF cell counts. Numbers of macrophages, lymphocytes, neutrophils and eosinophils were scored using light microscopy [21].

### Preparation of lung homogenates

Lung samples were homogenized in 1 % Triton X-100 (Sigma-Aldrich)/PBS containing protease inhibitor (Complete Mini, Roche Diagnostics, Germany) using a Precellys 24 tissue homogenizer (Bertin Technologies, France). Homogenates were centrifuged at 14,000 rpm for 5 min, and supernatants were collected. The protein concentration of each sample was assayed using the Pierce BCA protein assay kit standardized to BSA according to the manufacturer’s protocol (Thermo Fisher Scientific, USA). The homogenates were diluted to a final concentration of 1 mg protein/mL [22, 23].

### Ex vivo lung restimulation with house dust mite

Lung cell suspensions were prepared by cutting the lung into small pieces and by adding digestion buffer, containing DNase I and Collagenase A (Roche Diagnostics, Germany), for 30 min. The digestion was stopped using fetal calf serum (FCS; Hyclone Laboratories, USA). The lung pieces were transferred toward a 70-µm nylon cell strainer (BD Biosciences, The Netherlands) and rinsed with 10 mL RPMI. Cells were washed and resuspended in RPMI-1640 culture medium (Lonza, USA) supplemented with 10 % heat-inactivated FCS and 0.1 % penicillin–streptomycin solution (Sigma-Aldrich). Total number of cells was calculated using a Beckman Z1 coulter^®^ Particle Counter (Beckman, USA). Lung cells (4 × 10^5^ cells/well) were cultured in a Greiner bio-one CellSTAR 96-well U-bottom plate (Greiner Bio-One B. V., The Netherlands) in medium with or without 50 µg/mL HDM (Greer Laboratories, USA). The supernatant was harvested after 4 days of culture at 37 °C in 5 % CO_2_ and stored at −20 °C until further analysis [13].

### Measurement of cytokines

A standard Th1/Th2/Th17 assay (BD Biosciences, The Netherlands) was used to determine cytokine concentrations in lung homogenates and supernatants of lung restimulation according to the manufacturer’s instructions. IL-33, CCL17, CCL20 and CCL22 were measured with a DuoSet ELISA (R&D Systems), and IL-13 and IL-5 with a Ready-SET-Go!^®^ ELISA (eBioscience, USA). The concentrations of these cytokines were expressed as pg/mg protein in lung homogenates and pg/mL in BALF and restimulation supernatants.

### Statistical analysis

Results are presented as mean ± standard error of mean (SEM). Data were statistically analyzed by one-way ANOVA and post hoc Bonferroni’s multiple comparisons test. *P* < 0.05 were considered significant. Statistical analyses were conducted using GraphPad Prism software (version 6.04).

## Results

### Dietary intervention with FF*BB* reduced pulmonary eosinophilic inflammation in HDM-allergic mice

Airway hyperresponsiveness (AHR) to methacholine aerosols was measured at baseline; no differences were observed between the PBS- and HDM-challenged mice fed the different diets (Fig. 1b). In the PBS-challenged mice fed the FF*BB* diet, the baseline airway resistance tended to increase compared to PBS mice fed the control diet (Fig. 1b); however, methacholine exposure did not significantly enhance hyperreactivity in these mice as compared to control diet fed mice (Fig. 1c). HDM-challenged mice showed a higher AHR response upon methacholine exposure than PBS-challenged mice, but this did not reach significance (Fig. 1c). HDM challenge in GF*BB*- or FF*BB*-fed mice did not increase the AHR response compared to PBS-challenged mice fed the similar diet (Fig. 1c). Furthermore the AHR of HDM-challenged mice fed GF*BB* or FF*BB* remained below the AHR of HDM-challenged mice fed the control diet albeit this did not reach significance (Fig. 1c).

**Fig. 2:**
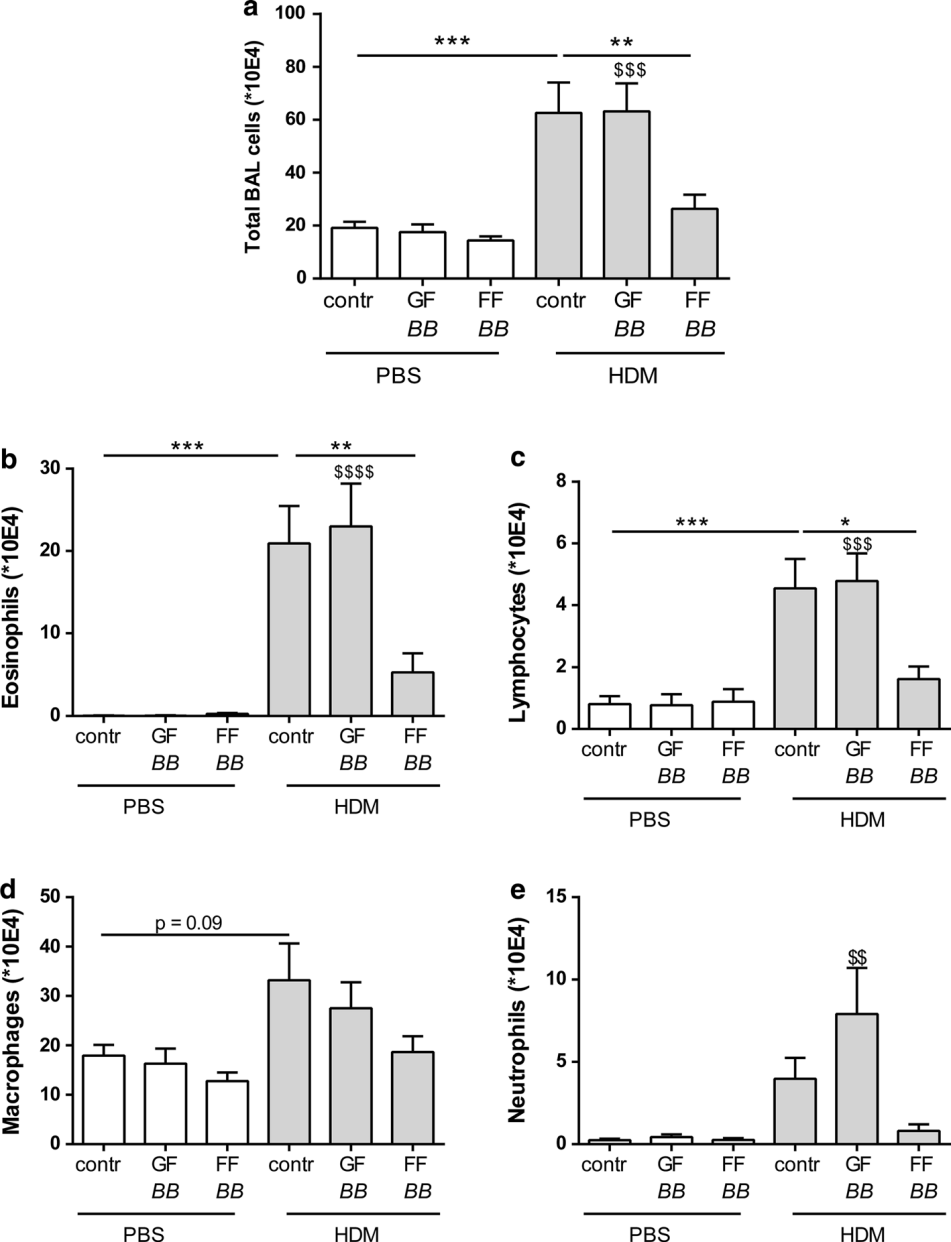
Dietary intervention with FF*BB* reduced pulmonary eosinophilic inflammation in the lungs of HDM-allergic mice. Differentiation of inflammatory cell infiltration in the BALF of house dust mite-allergic mice. HDM–PBS: HDM-sensitized and PBS-challenged mice (*white bars*), HDM–HDM: HDM-sensitized and HDM-challenged mice (*gray bars*), Contr: control diet, GF*BB*: mixture of GOS and long-chain fructo-oligosaccharide with *Bifidobacterium breve* M-16V diet, FF*BB*: mixture of short-chain fructo-oligosaccharide and long-chain fructo-oligosaccharide with *Bifidobacterium breve* M-16V diet. Total BALF cells (**a**), absolute number of eosinophils (**b**), lymphocytes (**c**), macrophages (**d**) and neutrophils (**e**). Results are shown as mean ± SEM. Statistical significance of differences by one-way ANOVA and post hoc Bonferroni’s multiple comparisons test. *P < 0.05, **P < 0.01, ***P < 0.001 compared to HDM–HDM;^ $$^ *P* < 0.01,^ $$$^ *P* < 0.001,^ $$$$^ *P* < 0.0001 compared to HDM–PBS group of corresponding diet, *n* = 6–9 mice/group

BALF was examined to investigate the inflammatory cell influx into the airways of HDM-allergic mice upon dietary intervention with control diet or the synbiotic diets (Fig. 2a). The total number of inflammatory cells in the BALF of HDM–HDM mice fed the control diet was significantly increased when compared to HDM–PBS mice (Fig. 2a). The HDM challenge induced an increase in the number of eosinophils and lymphocytes. The same tendency was shown for macrophages (Fig. 2b–d) compared to the PBS-challenged group. Dietary intervention with FF*BB* reduced the total number of inflammatory cells in HDM-allergic mice compared to HDM–HDM group fed the control diet (Fig. 2a); this reduction was mainly due to a decrease in the number of eosinophils and lymphocytes (Fig. 2b, c). Dietary intervention with GF*BB* did not suppress the total number of BALF cells (Fig. 2a) or the differentiated cell numbers (Fig. 2b–d). In this group, the number of neutrophils was significantly increased compared to the HDM–PBS control group fed the GF*BB* diet (Fig. 2e).

### Effect of synbiotics on inflammatory and Th2-type cytokines and chemokines in lungs of HDM-allergic mice

**Fig. 3:**
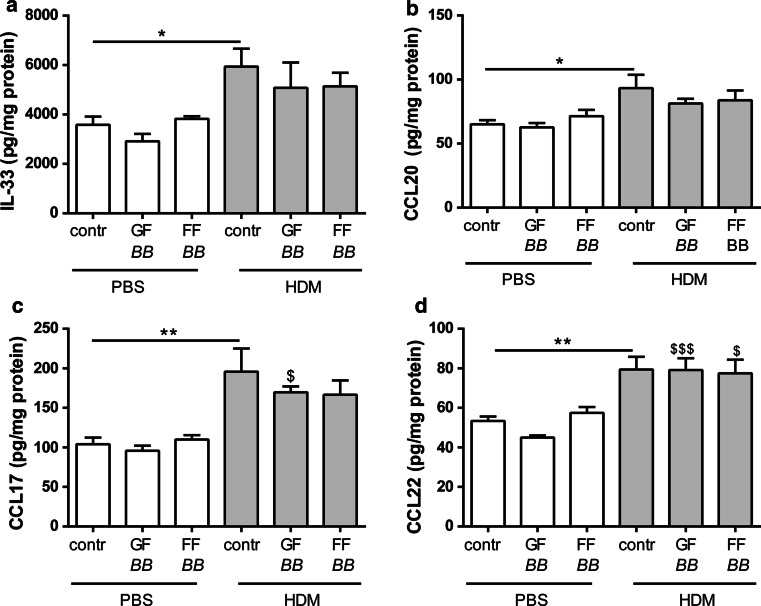
IL-33, CCL20, CCL17 and CCL22 in lungs of HDM-allergic mice. IL-33 (**a**), CCL20 (**b**), CCL17 (**c**) and CCL22 (**d**) concentrations were measured in supernatant of lung homogenates. HDM–PBS: HDM-sensitized and PBS-challenged mice (*white bars*), HDM–HDM: HDM-sensitized and HDM-challenged mice (*gray bars*), Contr: control diet, GF*BB*: mixture of GOS and long-chain fructo-oligosaccharide with *Bifidobacterium breve* M-16V diet, FF*BB*: mixture of short-chain fructo-oligosaccharide and long-chain fructo-oligosaccharide with *Bifidobacterium breve* M-16V diet. Results are shown as mean ± SEM. Statistical significance of differences by one-way ANOVA and post hoc Bonferroni’s multiple comparisons test. *P < 0.05, **P < 0.01 compared to HDM–HDM;^ $^ *P* < 0.05,^ $$$^ *P* < 0.001 compared to corresponding PBS group of diet, *n* = 5 mice/group

**Fig. 4:**
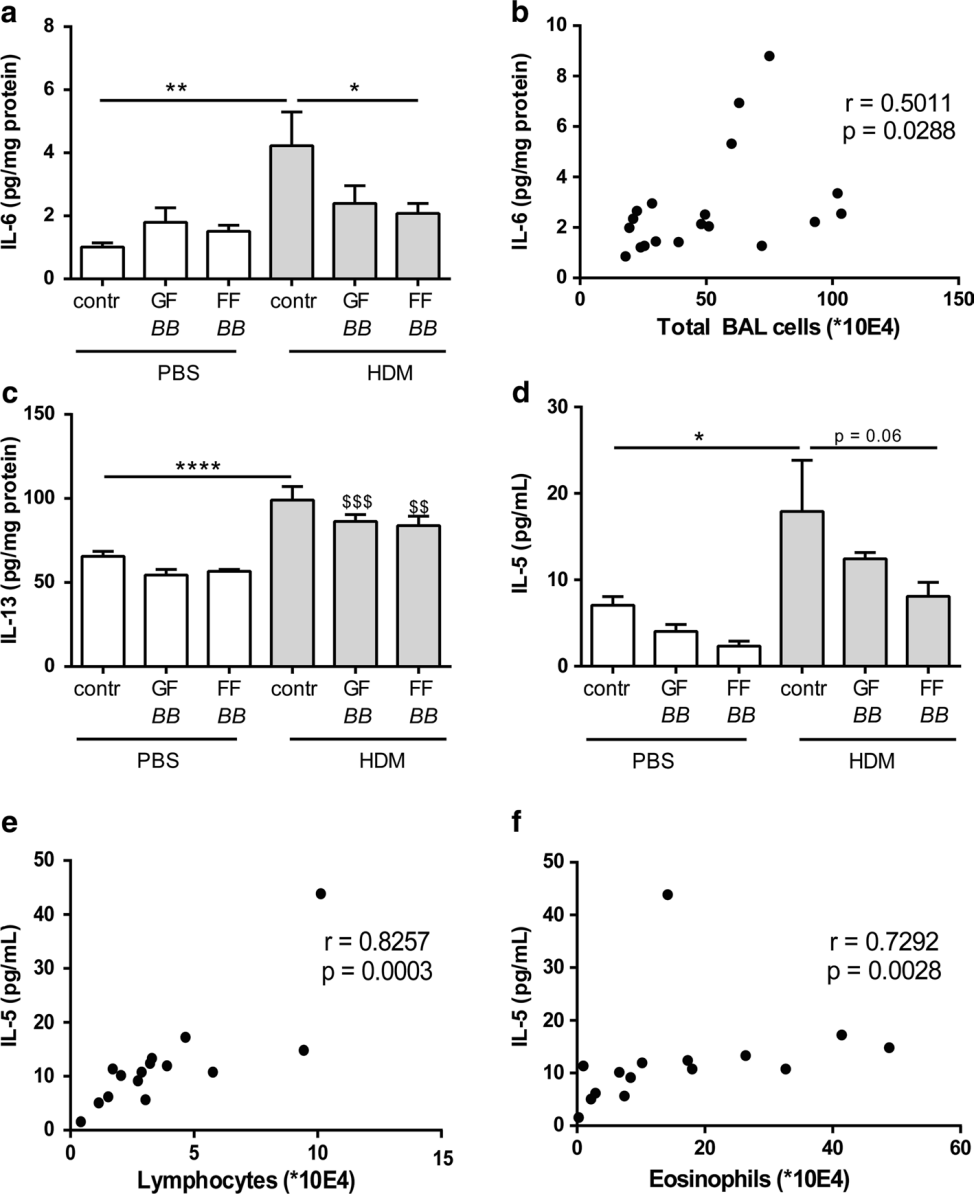
IL-6, IL-13 and IL-5 in lungs of HDM-allergic mice. IL-6 (**a**), IL-13 (**c**) concentrations were measured in supernatant of lung homogenates. IL-5 (**d**) was measured in the BALF. Correlation of IL-6 in lung homogenates and the total number of BALF cells (**b**), IL-5 in the BALF and the number of lymphocytes (**e**) and IL-5 in the BALF and the number of eosinophils (**f**). HDM–PBS: HDM-sensitized and PBS-challenged mice (*white bars*), HDM–HDM: HDM-sensitized and HDM-challenged mice (*gray bars*), Contr: control diet, GF*BB*: mixture of GOS and long-chain fructo-oligosaccharide with *Bifidobacterium breve* M-16V diet, FF*BB*: mixture of short-chain fructo-oligosaccharide and long-chain fructo-oligosaccharide with *Bifidobacterium breve* M-16V diet. Results are shown as mean ± SEM. Statistical significance of differences by one-way ANOVA and post hoc Bonferroni’s multiple comparisons test. *P < 0.05, **P < 0.01, ****P < 0.0001 compared to HDM–HDM;^ $$^ *P* < 0.01,^ $$$^ *P* < 0.001 compared to corresponding PBS group of diet, *n* = 5–9 mice/group. Correlation was analyzed using Spearman’s correlation test

IL-33, CCL20, CCL17 and CCL22 were significantly increased in supernatants of lung homogenates of HDM–HDM mice fed the control diet compared to the HDM–PBS control group. Dietary interventions with the different synbiotics did not affect these cytokines and chemokines (Fig. 3a–d). IL-6 and IL-13 concentrations in the supernatant of the lung homogenates and IL-5 in BALF of HDM–HDM mice fed the control diet showed a significant increase compared to the HDM–PBS control group (Fig. 4a, c, d). Dietary intervention with FF*BB* significantly decreased the HDM allergy-induced increase in IL-6 (Fig. 4a). IL-6 concentrations in lung tissue correlated positively with the total number of BALF cells (Fig. 4b, *r* = 0.5011, *P* = 0.0288). Dietary interventions with the different synbiotics did not affect this HDM-induced increase in pulmonary IL-13 (Fig. 4c), but tended to decrease the concentrations of IL-5 in the BALF (Fig. 4d). IL-5 concentrations in the BALF were positively correlated with the number of lymphocytes (Fig. 4e, *r* = 0.8257, *P* = 0.0003) and the number of eosinophils in the BALF, respectively (Fig. 4f, *r* = 0.7292, *P* = 0.0028).

### Synbiotic diets suppress T cell activity in HDM-allergic mice

**Fig. 5:**
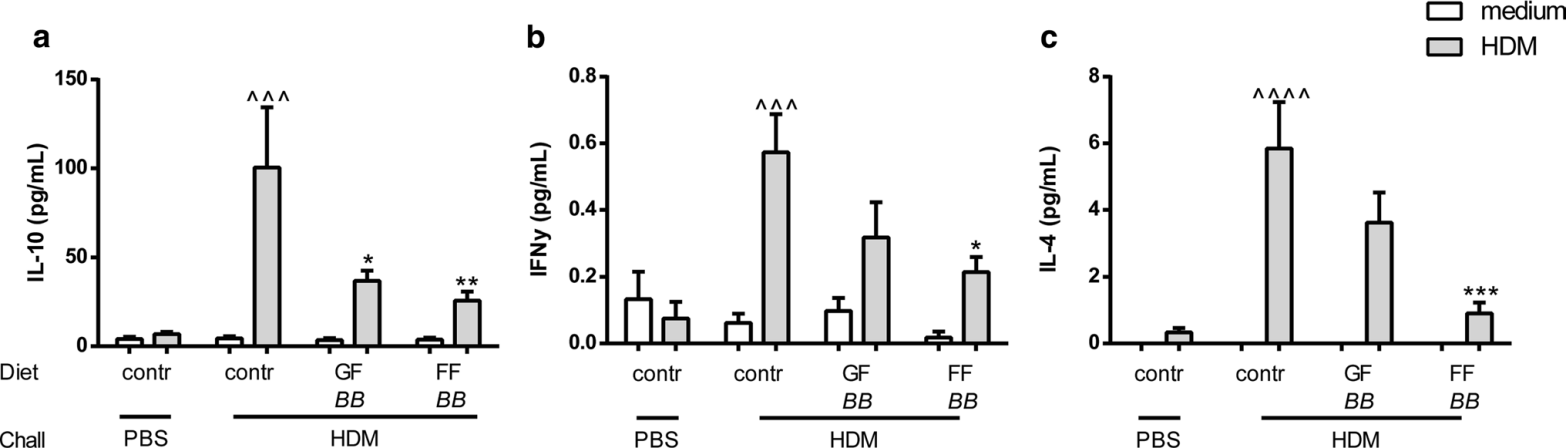
Synbiotic diets suppress antigen-specific T cell activation in HDM-allergic mice. Lung cell suspensions were ex vivo restimulated with HDM for 4 days (37 °C, 5 % CO_2_). IL-10 (**a**), IFNγ (**b**) and IL-4 (**c**) concentrations were measured in the supernatant. HDM–PBS: HDM-sensitized and PBS-challenged mice (*white bars*), HDM–HDM: HDM-sensitized and HDM-challenged mice (*gray bars*), Contr: control diet, GF*BB*: mixture of GOS and long-chain fructo-oligosaccharide with *Bifidobacterium breve* M-16V diet, FF*BB*: mixture of short-chain fructo-oligosaccharide and long-chain fructo-oligosaccharide with *Bifidobacterium breve* M-16V diet. Results are shown as mean ± SEM. Statistical significance of differences by one-way ANOVA and post hoc Bonferroni’s multiple comparisons test. *P < 0.05, **P < 0.01, ***P < 0.001 compared to HDM–HDM; ^^^^^ *P* < 0.001, ^^^^^^ *P* < 0.0001 compared to HDM–PBS, *n* = 6–7 mice/group

To investigate the effects of different synbiotics on allergen-specific cytokine secretion of lung tissue, cell suspensions were ex vivo restimulated with HDM. The concentrations of IL-10, IFNγ and IL-4 were significantly increased upon HDM stimulation of lung cells of the HDM–HDM mice fed the control diet, whereas HDM–PBS mice showed no increase compared to medium stimulation (Fig. 5a–c). Dietary intervention with GF*BB* and FF*BB* significantly decreased the concentrations of IL-10, while FF*BB* also decreased IL-4 and IFNγ (Fig. 5a–c). Although upon HDM stimulation IL-17A concentrations were increased in the lung cells of HDM–HDM mice fed the control diet, both dietary interventions showed no effect (data not shown).

## Discussion

This is the first study in which anti-inflammatory effects of different oligosaccharide mixtures (e.g., GF or FF) in combination with *Bifidobacterium breve* M-16V (*BB*) (synbiotics) were investigated on the development of HDM-induced allergic inflammation in a murine model appropriate to mimic human allergic asthma. To mimic the human features of asthma, murine models for HDM-allergic asthma are commonly used, since HDM is one of the most common allergens associated with human allergic asthma [24, 25]. In this study, the effects of the synbiotic diets on AHR remain unclear; by contrast, strong protective effects on airway inflammation were observed in HDM-allergic mice. Total inflammatory BALF cell numbers were significantly increased in HDM–HDM-allergic mice when compared to HDM–PBS control mice. In contrast to GF*BB*, dietary intervention with FF*BB* significantly decreased the total inflammatory cell number. This decrease was mainly due to the reduction in the number of eosinophils and lymphocytes compared to HDM–HDM mice fed the control diet. Although FF*BB* was not able to reduce epithelial and DC-related cytokines (IL-33) and chemokines (CCL20, CCL17 and CCL22) known to contribute to allergic sensitization, FF*BB* decreased the pro-inflammatory cytokine IL-6. IL-6 was found to correlate with the number of total inflammatory cells in the BALF. Furthermore, FF*BB* reduced Th2-related cytokines (IL-4 and IL-5) and Th1-related cytokine IFN-γ concentrations in lung homogenates or in supernatants of ex vivo restimulated lung cell suspensions. GF*BB* was less effective but showed a similar pattern with regard to these parameters, and both FF*BB* and the GF*BB* diet reduced IL-10 concentrations after ex vivo restimulation of lung cells with HDM.

The data show that in particular the FF*BB* diet was capable of suppressing airway inflammation, whereas it did not suppress Th2-driving mediators such as IL-33, CCL17 and CCL22. Epithelial cells are known to secrete IL-33 upon HDM stimulation, which activates DC [26] and is a chemoattractant for Th2 cells [27, 28]. IL-33 has also been shown to be increased in bronchial biopsies of asthmatic patients compared to non-asthmatic patients [29]. In the present study, a significant increase in IL-33 concentrations was observed in lung homogenates obtained from HDM-allergic mice and no evidence was found that the dietary interventions modulated this increase.

Another relevant chemokine that is produced by the epithelium is CCL20 which is responsible for the attraction of immature DC to the lung [26, 30]. From human studies, it is known that CCL20 levels are increased in asthmatic patients compared to healthy controls, which is even more pronounced after allergen challenge [31]. In the current study, a significant increase in CCL20 in lung homogenates of the HDM–HDM mice compared to the HDM–PBS mice fed the control diet was found, which remained unaffected by the synbiotic diets.

In patients suffering from atopic asthma, it was shown that after a challenge with HDM, the concentrations of CCL17 were also increased in the BALF [32]. CCR4 and its ligand CCL17 are up-regulated in the airways of asthmatic patients after challenge and contribute to the Th2 cell recruitment in asthma [32, 33]. However, CCR4 is not only a ligand for CCL17, but it also binds CCL22 with an even higher affinity [34]. CCR4 is expressed by regulatory T cells, mast cells and Th2 cells and is known to play a pivotal role in allergic diseases. In bronchial biopsies of asthmatic patients, most of the T lymphocytes were CCR4 positive [35]. In the current study, a significant increase in CCL17 and CCL22 was found in lung tissue of the HDM–HDM mice compared to the HDM–PBS mice fed the control diet. However, the synbiotic diets did not affect these pulmonary concentrations of CCL17 and CCL22. Overall, these results indicate that although the FF*BB* diet effectively reduced lung eosinophilia and lymphocyte influx, the diet did not suppress release of Th2-polarizing mediators by epithelial cells and/or DC.

IL-6 is produced not only by inflammatory cells such as DC and macrophages, but also by lung epithelial cells after stimulation with an allergen. The murine model for HDM-induced allergic asthma showed elevated pulmonary IL-6 concentrations, which is in line with human studies that showed elevated IL-6 levels in BALF, sputum and serum from asthmatic patients [36, 37]. Dietary intervention with FF*BB* significantly decreased the concentration of IL-6 in lung homogenates, which correlated with the observed decrease in the influx of inflammatory cells. Hence, the decrease in IL-6 by the FF*BB* diet may relate to the protective effect of this diet on lung inflammatory responses as seen in this animal model.

Besides the pro-inflammatory IL-6, IL-13 and IL-5 are also investigated. These allergy-driven cytokines were secreted by antigen-specific Th2 cells and mast cells as well as by ILCs. IL-13 is also capable of triggering eosinophils and macrophages [38], and IL-5 is a Th2 cytokine which is essential for the maturation and activation of eosinophils [39]. The concentration of IL-13 in lung tissue and IL-5 in the BALF was significantly increased in HDM-allergic mice compared to control mice. This effect corresponds to human studies which showed that IL-13 levels were increased in sputum of asthmatic patients [38, 40, 41]. Furthermore, IL-5 mRNA levels are increased in bronchial biopsies of asthmatic patients compared to healthy controls [42]. Since IL-5 is essential for eosinophil maturation and eosinophils are important in asthma, animal and human studies showed that eosinophilia in blood and BALF can be reduced with monoclonal antibodies against IL-5 [39, 43]. Although FF*BB* did not reduce the concentration of IL-13 in lung homogenates, it showed a strong tendency to decrease the concentration of IL-5. Moreover, IL-5 in BALF was positively correlated with eosinophil and lymphocyte numbers. The reduction in inflammatory cell influx by FF*BB* may be a consequence of mechanisms involving the suppression of challenge-induced IL-6 and IL-5 secretion by lung inflammatory cells.

The FF*BB* diet suppressed lymphocyte influx, and indeed ex vivo antigen-specific restimulation of lung cell suspensions showed reduced Th1 (IFNγ)- and Th2 (IL-4)-type cytokine release by lung cells of HDM-challenged mice fed the FF*BB* diet. IFNγ is a Th1 cytokine which is found to be increased in BALF and peripheral blood of asthmatic patients. In mouse models, IFNγ is increased at mRNA levels in the airway epithelium [44]. IL-4 is one of the key players in the development of allergic asthma. A high IL-4 milieu is necessary for the differentiation of Th2 cells that in turn can also produce IL-4 upon antigen binding [26]. In patients with asthma, increased IL-4 protein levels were found in BALF and serum [45]. When patients with mild asthma were nebulized with IL-4, an elevation of eosinophil number in the sputum was observed. In addition, it was shown that after nebulizing with anti-IL-4 antibodies, eosinophilic inflammation was reduced in mice [46]. Since both allergen-induced IFN-γ and IL-4 were decreased after restimulation ex vivo with HDM in the FF*BB* group, these results support the hypothesis that the influx of inflammatory cells into the lung tissue upon the previous in vivo allergen challenge was reduced by the diet.

The FF*BB* diet may be capable of reducing the allergen-specific effector response, which can include regulatory cytokines such as IL-10. Upon ex vivo HDM stimulation, IL-10 was also decreased in FF*BB*-fed group. Besides, FF*BB* also GF*BB* reduced IL-10, whereas IFN-γ and IL-4 were not significantly lowered by GF*BB*. This may imply that GF*BB* acts via a different mechanism of action in particular targeting HDM-induced increase in IL-10 or that FF*BB* is more effective in dampening the inflammatory cascade.

**Fig. 6:**
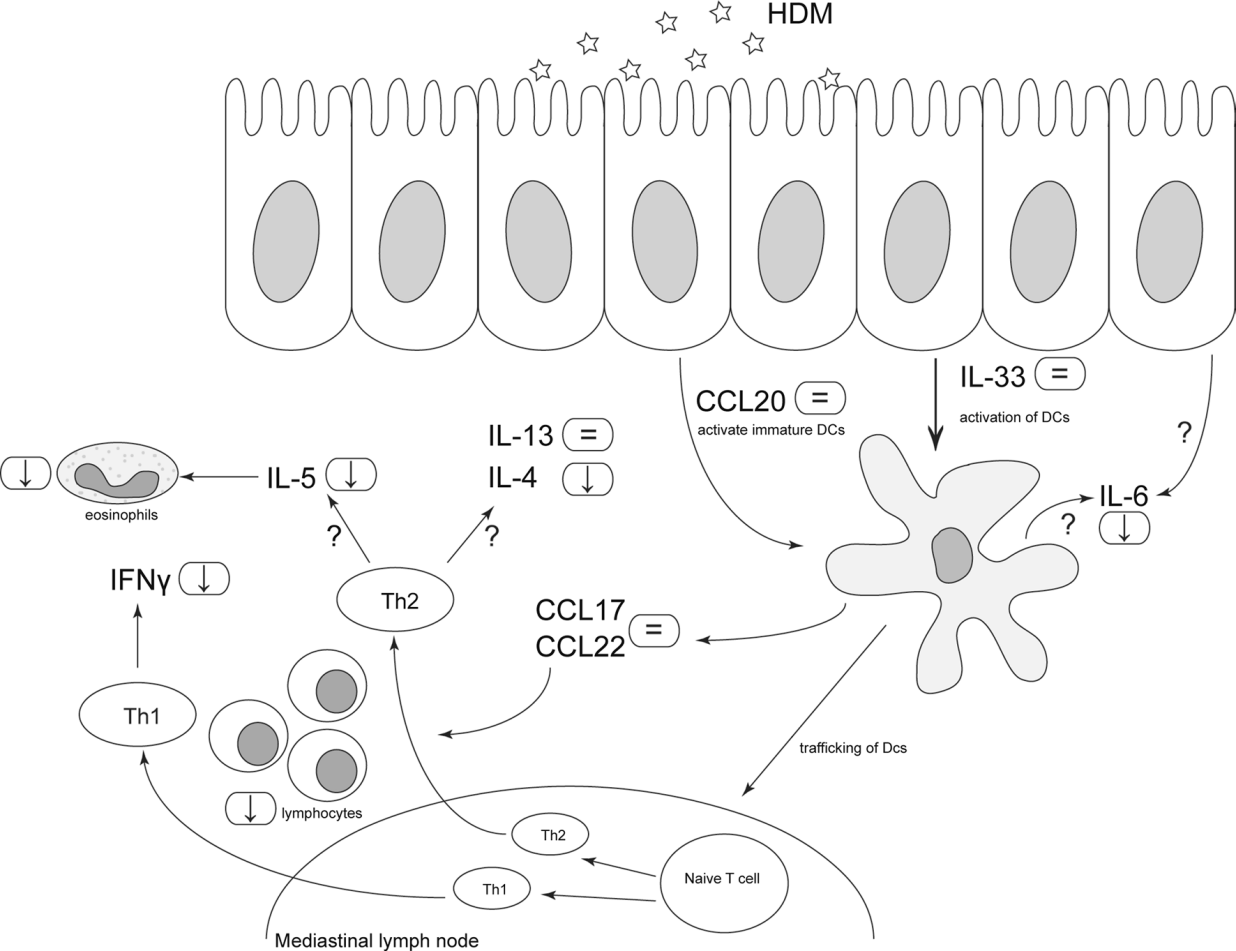
Overview of the effects of the synbiotic diet FF*BB*. After the initial exposure to HDM, CCL20 is secreted by the airway epithelium, which will activate immature DCs. IL-33, also secreted by the epithelium, activates the DCs. Both CCL20 and IL-33 concentrations in lung homogenate supernatants were not affected by the FF*BB* diet. Activated DCs secrete CCL17 and CCL22, which are chemo-attractants for Th2 cells, and will traffic to the mediastinal lymph nodes to differentiate naïve T cells into Th2 cells. The FF*BB* diet did not affect CCL17 and CCL22 release by these DC. In contrast, IL-6, which can be secreted by lung epithelial cells or inflammatory cells such as DC, was suppressed which correlated with the reduced inflammatory cell influx. Mainly lymphocyte and eosinophil influx was lowered by the FF*BB* diet. Among other cells, Th2 cells secrete IL-13, IL-4 and IL-5 and Th1 cells secrete IFN-γ. Although IL-13 remained high, the FF*BB* diet reduced IL-4 and IFNγ. Furthermore, FF*BB* tended to suppress IL-5 levels which correlated with the reduced number of lymphocytes and eosinophils upon dietary intervention

Hence, the current study shows a protective effect of dietary FF*BB* in HDM-induced airway inflammation in mice when provided before and during allergic sensitization and challenge. FF*BB* did not affect IL-33 and CCL20 induction, which are known to initiate the development and activation of CCL17- and CCL22-producing Th2-polarizing DC contributing to allergic sensitization. However, FF*BB* may have reduced the influx of eosinophils and allergen-specific T cells by suppressing local secretion of IL-6 and IL-5 by inflammatory cells via currently unknown mechanisms (Fig. 6).

Despite the strong effect of the FF*BB* diet on airway inflammation, it remains to be assessed whether FF*BB* may improve the clinical outcome (asthma) since the effects of FF*BB* on the AHR remained unclear in the current study. Future studies are needed to further optimize the effectivity of FF*BB* in prevention and/or treatment of HDM-induced asthma. This may include and earlier introduction prior to sensitization and dose optimization. Furthermore, combining FF with other, bifidobacteria or lactobacillus strains may be considered. Indeed in other mice studies, positive effects of *L. rhamnosus* have been observed on AHR and lung inflammation [13, 47]. A combination of FF with this or other bacteria might have beneficial effects on allergic asthmatic features. The mode of action of FF*BB* in suppressing lymphocyte and eosinophil influx hereby dampening the allergic asthma pathway is currently unclear. We hypothesize that compared to GF*BB*, FF*BB* changes the microbiota in a different manner and via these changes differentially affects the mucosal and systemic immune response. One of the ways by which this may occur is via altering the levels or patterns of short-chain fatty acids (SCFA) produced upon bacterial fermentation of the oligosaccharides in the intestine. These SCFA become available in the blood stream and have anti-inflammatory properties by acting via the GPR41 and GPR43 receptors and are known to suppress airway inflammation [48]. Indeed, SCFA have been shown to dampen asthmatic responses in HDM-allergic mice fed a high-fiber diet by lowering the number of eosinophils and lymphocytes and the concentration of Th2-related cytokines [49]. However, whether this also underlies the mechanism of FF*BB* needs to be further elucidated. The promising results of our study suggest the potential clinical application of this intervention, and in future studies the use of FF*BB* as an adjunct therapy for budesonide treatment, a corticosteroid used to treat asthma in humans, may be considered as well.

In conclusion, dietary intervention with a synbiotic supplementation can suppress pulmonary inflammation in HDM asthmatic mice, although the FF*BB* showed a more beneficial effect on the management of HDM-induced allergic asthma compared to GF*BB*. The mode of action of FF*BB* in suppressing airway eosinophilia and lymphocyte numbers hereby dampening the allergic asthma pathway needs to be further elucidated, but the promising results of our study suggest the potential clinical application of this intervention.

## Abbreviations

AHR: Airway hyperresponsiveness
BALF: Bronchoalveolar lavage fluid
*BB*: *Bifidobacterium breve* M-16V
DC: Dendritic cell
FF*BB*: Short-chain and long-chain fructo-oligosaccharide with *Bifidobacterium breve* M-16V
FOS: Fructo-oligosaccharide
GF*BB*: Galacto-oligosaccharide and long-chain fructo-oligosaccharide with *Bifidobacterium breve* M-16V
GOS: Galacto-oligosaccharide
HDM: House dust mite
i.n: Intranasally
ILC2: Innate lymphoid cell type 2
R_L_: Lung resistance
